# Efficacy of Short–*Term *Anxiety-Regulating Psychotherapy on Love Trauma Syndrome

**Published:** 2011

**Authors:** Mahmood Dehghani, Mohammad- Kazem Atef-Vahid, Banafsheh Gharaee

**Affiliations:** 1 PhD student of Clinical Psychology, Mental Health Research Center and Tehran Psychiatric Institute, Iran University of Medical Sciences. Shahid Mansoori Alley, Niayesh St., Sattarkhan Ave., Tehran, Iran, IR. Tel.; 2 Assistant Professor of Clinical Psychology, Mental Health Research Center and Tehran Psychiatric Institute, Iran University of Medical Sciences, Tehran, Iran.

**Keywords:** Depression, Love Trauma Syndrome, Short Term Dynamic Psychotherapy

## Abstract

**Objective:** This study has been conducted to investigate the efficacy of short-term dynamic psychotherapy on Love Trauma Syndrome (LTS) in female students. LTS includes a constellation of intensive signs and symptoms which appear following the breakdown of a romantic relationship after a long time. It interrupts person's function in many areas (academic, social or professional) and leads to maladaptive reactions.

**Methods:** This study was a multiple baseline single-case experimental study. The sample included five cases who were selected based on purposeful sampling procedure. The intervention was conducted based on McCullough's manual for short-term dynamic psychotherapy. In general, the study consisted of three stages including the baseline evaluation, the intervention period, and follow-up. The instruments included the Love Trauma Inventory (LTI), Beck Depression Inventory (BDI-II), Global Assessment Function) GAF) of DSM-IV and Millon Personality Inventory MCMI-II the data were analyzed using the clinical significance method and the recovery percent formula.

**Results:** All subjects who completed the treatment showed significant improvement in their symptoms including depression and general functioning.

**Conclusion:** It appears that McCullough's short term anxiety-regulating psychotherapy is effective in treating LTS.

## Introduction

Love and grief are two sides of the same coin; we cannot have one without risking the other. Only by understanding the nature and pattern of love we can begin to understand the problem of grief. Conversely, loss of a love can teach us much about the nature of love. While love is the most profound source of pleasure in our lives, loss of those whom we love is the most profound source of pain. However, loss is the common consequence of love and grief is the price that must be paid (1).

People experience love and grief throughout their lives. It seems that a significant loss is re-enacting of the separation-individuation process. In fact, grief takes us to our core (2). Freud had once said: "finding an object is in fact re-finding of it" (3). In the same way, today we can reasonably claim that losing an object is in fact re-losing of it. Harvey and Miller (4) believed that loss and grief can occur without death.

We can find one of the most common and intensive grief in the breakdown of a romantic relationship. A fact that is well-known and tangible in literary writings, whereas it is vague and unknown in the scientific literature of grief and loss. This is in spite of the fact that the loss and grief in romantic relationship is similar to other losses (4,5), yet it has been generally neglected by the researchers in the field. Although Harvey and Miller (4) stated that loss and grief are generally related to death, loss and grief in romantic relationship refers to the termination of a relationship by one or both of them.

Horowitz (6) described how after the loss of a love the mourner's "schemas" of his or her important relationships come into conflict. The person wants to hold onto the old schemas in which the beloved object is present. He or she is also confronted with the fact that the beloved object is gone and is no longer available.

According to Horowitz (6), mourning involves a "working through" process in which the individual's schemas of self and others come into line with the demands of reality, and the individual is able to accept a new image of his or her present situation. After a loss, the mourner must reevaluate his or her own role in the relationship as well as that of the partner or love object. This reevaluation should lead to a sorting-out process, in which certain aspects of the experience are assigned to the self and some to the lost object.

Whereas the physical absence of a dead beloved object inevitably helps the mourner with believing the loss, mental absence of a beloved object in his or her physical presence in real world in romantic loss makes this condition more complicated. It seems that the physical presence of the beloved object in real world is a gleam in romantic loss which can remain alive to the end of life. Phantasy and physical presence of the beloved object may be fundamental in making the romantic loss lasting and complicating.

In healthy mourning, some of the functions of the internal object are gradually taken over by new relationships with new objects in the external world, yet there are aspects of the internal relationship with the deceased that remain unique. The self is never again the same as it was in that relationship, and the object is found to be unique in ways that cannot be fully replaced, because of the core of individuality, of uniqueness of the self and object representations (7).

Stroebe et al (8) described and compared the "modernist approach", which emphasizes achieving autonomy by breaking off emotional bonds with a deceased beloved object with (b) the "romantic approach" which emphasizes preserving such bonds as essential aspects of identity and personal meaning. The romantic approach provides an additional dimension because this approach sees the mourning as a process of balanced object relating, not simply as a detachment.

Richard Rosse (9) introduced love trauma syndrome (LTS) for the first time. LTS includes a constellation of severe signs and symptoms which appear after the breakdown of romantic relationship after a long time. It interrupts person's function in many areas (academic, social or professional) and leads to maladaptive reactions. Rosse (9) introduced Love Trauma Inventory (LTI) as a scale for assessing the intensity of love trauma.

Love is one of the most wonderful emotions we can experience, but it can also be very painful. The emotional pain of a broken heart can be manifested as LTS. The syndrome exists as a discrete clinical entity with its own unique constellation of symptoms. Previously, love traumas were thought simply to precipitate other common psychiatric conditions such as depression or "adjustment disorders." However, these generic psychiatric disorders are not adequate for capturing the unique character of the condition that occurs following a love trauma. LTS is commonly complicated by a variety of other psychiatric conditions such as depression and substance or alcohol abuse (9).

The essential feature of LTS is the development of symptoms after experiencing a love trauma. Patients initially experience the love trauma as a "shock". What contributes to the sense of shock is that the love trauma violates the person's desired expectations of the relationship, and assumptions of safety in the relationship. Love trauma is experienced as a severe stress and is traumatic in some way. Rosse (9) means that the person experiences a significant emotional, psychological, or physical distress by "traumatic". Four significant criteria Arousal is associated with symptoms of anxiety such as irritability and sleep disorders. Avoidance includes attempts to avoid exposure to any cues that remind the person of love trauma. Automatic remembering involves the intrusive thoughts and memories common in LTS. Emotional anaesthesia refers to the decreased ability of some people with LTS to be able to experience love feelings in the future. Significance of someone's love trauma is also determined by considering its pervasive, persistent, and impairment effects on person's life (9).

In spite of the fact that this kind of grief is common, particularly in adolescence and youth, there is a dearth of research on the breakdown of a romantic relationship and its consequences. Breakdown of romantic relationships is similar to other losses and leads to grief (4), which in some cases can have intensive or extensive consequences in life. Luquet (5), Parkes (1), Harvey and Miller (4) emphasized the significance of the loss of love and its consequences.

When people are confronted with a loss, their reliance on serious longstanding and defensive mechanisms disrupts grieving process. According to O'Neil and Keane (2), crying itself is necessary. Otherwise, the emotions will build up and burst out in another way. "Sorrow that has no vent in tears makes other organs weep". Hence, there must be a purpose in physical and emotional responses to loss. Emotional tears have practical functions, which help the body to alleviate suffer and cleanse grief's toxins. Grief takes us to our core. It is not unique to human. Animals can also be heard howling when they experience the death of one of their own. Grieving is an instinctive primitive reaction.

The goal of this study was to examine the efficacy of short-term anxiety-regulating psychotherapy in improving the signs and symptoms of love trauma.

## Materials and Methods

*Participants and procedure*

Statistical population included female students who came to counseling clinics at three universities of Tehran. These students had experienced a serious collapse in the romantic relationship more than three months ago. All cases were referred by psychologists to these clinics. Five students who met the inclusion criteria were selected from this population based on purposeful sampling. Inclusion criteria were as follows: 1) 20–30 years of age, 2) negative history of intercourse, 3) at least a six months lasting relationship, 4) no mutual agreement on terminating the relationship, 5) the goal of marriage, 6) consulting three months after the termination, 7) GAF scores of 50 to 61, and 8) negative history of medication or any form of therapy. Exclusion criteria were serious clinical syndromes or personality disorders (such as psychosis or BPD) and serious suicidal idea or plan.

Millon Personality Inventory MCMI-II, Global Assessment Function (GAF) Scale of DSM-IV, Love Trauma Inventory (LTI) and clinical interview were used for selecting these five cases. All of the subjects were informed of the procedures and the baseline evaluations began when they gave their consent.

The treatment method used in this study was the short term anxiety-regulating psychotherapy. This is an integrative short-term dynamic psychotherapy model introduced by McCullough and his colleagues (10). This model utilizes an empathic and collaborative relationship with restructure defenses and anxieties that block feelings relating with self and others. This model retains many fundamental components of prior psychodynamic psychotherapies (11). McCullough's manual for short-term dynamic psychotherapy includes three main parts; defense restructuring, affect restructuring and self and other restructuring (10). Intervention in the present study included 20 sessions based on McCullough's manual, called "Treating Affect Phobia". Intervention also included baseline evaluation and a two-month follow-up.

*Measures*

BDI-II is a revised form of BDI which has been developed for assessing the intensity of depression. BDI-II included 21 items. The BDI-II is a revised form of BDI which has been developed for assessing the intensity of depression. BDI-II included 21 items. The reliability and validity of the BDI-II have been well established, with a test-retest reliability coefficient of 0.93 and internal reliability of 0.86. Internal consistency for psychiatric outpatients and college students were 0.92 and 0.93 respectively (12). A Cronbach's alpha of 0.91 and a test-retest reliability of 0.94 were reported by Fata, Birashk, Atef-Vahid and Dabson in a study from Iran (13). In this inventory the cutoff point is 17.

MCMI-II is the revised form of Millon Clinical Maltiaxial Inventory-I. MCMI-II was developed by Theodor Millon (14). This instrument is based on his biopsychosocial theory. The inventory was standardized in Iran by Khage-Mugahi et al (15). The Mode of the Kuder-Richardson coefficients for all scales was 0.85, and the mean was 0.80. Besides, the mode of the test-retest reliabilities of the scales was 0.86.

GAF is one of DSM-IV scales (16), in which the clinician evaluates the patient's global function during a specific time based on his or her clinical judgment. Function includes three main areas including psychological, social and professional aspects. This scale covers a spectrum of health with 10 interval points (from 0 to 100).

Rosse (9) introduced LTI in order to assess the severity of love trauma. It consists of 10 items and each item includes four choices. LTI shows how emotionally injured you have been by past love trauma events. In a student sample, Cronbach's alpha was 0.81 and reliability by test, re-test with one week interval was reported to be 0.83 (17). In this inventory the cutoff point is 20.

*Data analysis methods*

Methodology used in this research was a multiple baseline single-case experimental study. Two methods, the clinical significance (18) and the recovery percent formula (19) were used for data analysis. When individual's scores in target problems decrease to the normal level, i.e., below the cutoff point, it can be concluded that the results are clinically significant (18). Recovery percent formula is one of the methods used for assessing clients' progress. Blanchard and Schwars introduced this formula, and considered more than 50% recovery to be clinically significant (18).

## Results

All of the subjects were female with their age ranging from 24 to 26 years. Three of them were undergraduate and the others were graduate students. One of the subjects dropped out during therapy. Descriptive data are shown in table 1.

**Table 1:** Descriptive data of the clients

Clients	Age	Education	Duration†	Termination‡
First	25	MSc §	12 Months	3 Months
Second	26	MSc	18 Months	6 Months
Third	24	BSc ||	10 Months	3 Months
Fourth	25	BSc	14 Months	7 Months
Fifth	24	BSc	8 Months	3 Months

† the time they were in relationship,

‡ the time between the end of relationship and baseline stage of intervention,

§ Master of Science,

|| Bachelor of Science

**Figure 1:**
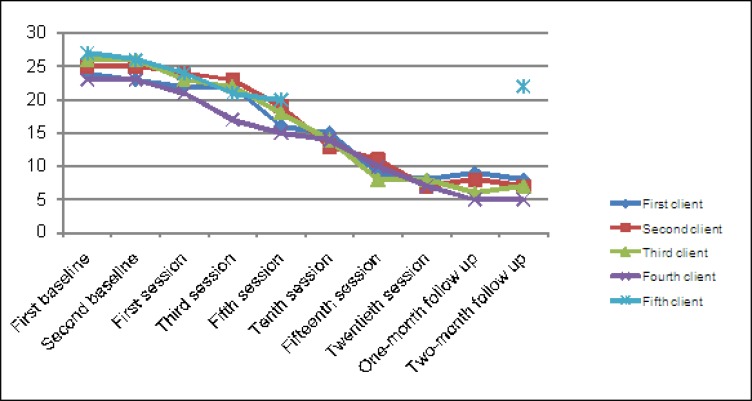
Love trauma inventory profile

Table 2 and figure 1 show the recovery index based on LTI. The recovery index was 63% for the first client, 70% for the second client, 65% for the third client, and 66% for the fourth client. Besides, recovery index at two months of follow-up was 63% for the first client, 70% for the second client, 69% for the third client, and 76% for the fourth client. Recovery index for the fifth client was 8% at follow-up stage.

**Table 2:** Love trauma inventory scores

LTI Scores	First client	Second client	Third client	Fourth client	Fifth client
First baseline	24	25	26	23	27
Second baseline	23	25	26	23	26
First session	22	24	23	21	24
Third session	22	23	22	17	21
Fifth session	16	19	18	17	20
Tenth session	15	13	14	14	-
Fifteenth session	9	11	8	10	-
Twentieth session	8	7	8	7	-
Recovery	63%	70%	65%	66%	-
One month of follow-up	9	8	6	5	-
Two months of follow-up	8	7	7	5	22
Recovery	63%	70%	69%	76%	8%

**Figure 2:**
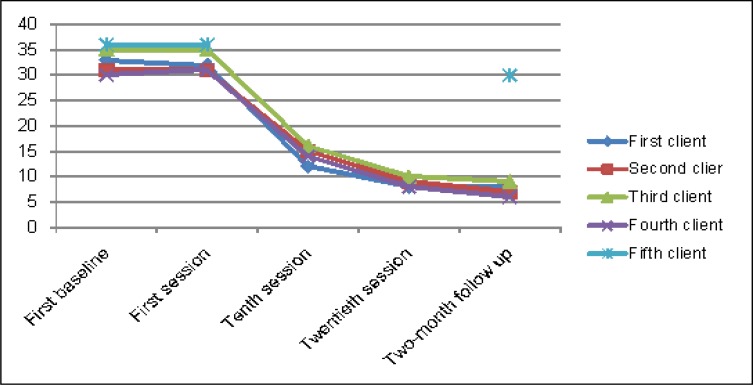
BDI**-**II profile

It is shown in table 3 (also in figure 2) that the recovery index based on BDI-II after the final session was 75% for the first client, 71% for the second client, 71% for the third client, and 74% for the fourth client. Besides, the recovery index at two-month follow-up was 75% for the first client, 78% for the second client, 74% for the third client, and 80% for the fourth client. However, it was 16% at follow-up stage for the fifth client.

**Table 3:** BDI**-**II scores

BDI-II	First client	Second client	Third client	Fourth client	Fifth client
Baseline session	33	31	35	30	
First session	32	32	35	31	36
Tenth session	12	15	16	14	-
Twentieth session	8	9	10	8	-
Recovery	75%	71%	71%	74%	-
Two months of follow-up	8	7	9	6	30
Recovery	75%	78%	74%	80%	16%

Table 4 shows clients' GAF scores before intervention, after intervention and during follow-up. The initial GAF scores for all clients was between 51 and 60 and it was between 71 and 80 after intervention and during follow-up. While the GAF for the fifth client remained between 51 and 60 during follow-up.

**Table 4:** GAF scores

GAF	First client	Second client	Third client	Fourth client	Fifth client
Baseline session	51-60	51-60	51-60	51-60	51-60
First session	51-60	51-60	51-60	51-60	51-60
Twentieth session	71-80	71-80	71-80	71-80	-
Recovery	36%	36%	36%	36%	-
Two- months of follow-up	71-80	71-80	71-80	71-80	51-60
Recovery	36%	36%	36%	36%	-

MCMI-II was used only as an instrument to screen serious personality disorders (such as BPD) or clinical syndromes (such as psychosis). All of the clients met the inclusion criteria for participation in the study based on the results of MCMI-II and clinical interview. However, all of them had some degrees of personality problems; especially the fifth client with some borderline traits. 

## Discussion

The main objective of this study was to examine the efficacy of short-term anxiety-regulating psychotherapy in treatment of LTS. Luquet (5) claims that collapse in the romantic relationships include series of stages, which are the same as the other griefs. Bowlby's (20) study of attachment and loss has also led to a broader theoretical perspective that emphasizes the importance of intimate attachments throughout the course of life. For the first time, Rosse (9) introduced LTS as a clinical focus, which includes a constellation of intensive signs and symptoms. LTS appears after the breakdown of a romantic relationship after a long time. It interrupts person's functions in many areas and leads to maladaptive reactions.

The findings of this study indicate the efficacy of the short term anxiety-regulating psychotherapy in treating LTS. Furthermore, these findings emphasize the importance of considering LTS as a clinical focus.

Ingram, Hayes and Scott (21) propose six indices for examining the efficacy of any psychological intervention. They believe that the results of interventional studies must be examined based on these indices including magnitude of change, universality of change, generality of change, acceptability, safety, and stability. The findings of this study are discussed according to these variables.

*1) Magnitude of change (degree of reduction in target symptoms):*

The results of LTI and BDI-II indicated a significant decrease in the severity of LTS and depression. When the scores of all clients who completed the treatment fell below the cutoff points following the intervention on both of these measures, it can be concluded that the changes are clinically significant (18,19). However, for the fifth client, the LTI and BDI-II scores remained higher than cutoff points at follow-up. Whereas GAF scores for all clients at the baseline stage were rated at 51-60 interval, after the intervention, the scores (except fifth client) were rated at 71-80 interval for all clients. However, the GAF score of fifth client placed her in 51-60 interval at follow-up. Although there is no cutoff point for GAF scale of DSM-IV, a 10-interval points of GAF is clinically significant. In other words, clients whose GAF fall between 71 and 80 are healthier than those in 51-60 interval.

*2) Universality of change (the percentage of change):*

The findings indicated that the target problems of all clients (except the fifth one) have been improved. LTI recovery index after final session indicated the recovery rate was 63% for the first client, 70% for the second client, 65% for the third client, 66% for the fourth client and 8% for the fifth. Furthermore, BDI-II recovery index after final session was 75% for the first client, 71% for the second client, 71% for the third client, 74% for the fourth client and 16% for the fifth client. Based on the recovery percent formula (18,19), more than 50% recovery is clinically significant. The Levels of GAF for all five clients at baseline stage fell in 51-60 interval, and after intervention and during follow-up, the GAF for all clients (except fifth client) fell in 71-80 interval. The GAF for fifth client, however, remained in 51-60 interval at the follow-up. Average of the recovery for all clients (except fifth client) was 36%.

*3) Generality of change(how much change have* *occurred in other areas)?:*

The findings indicated that GAF scores for the clients who completed the therapy increased from 51-60 to 71-81 interval. As GAF includes psychological, social and occupational areas, it can be concluded that this intervention improved the clients' functioning in various areas.

*4) Acceptability* *(rate of drop-out from therapy):*

Except for the last client, all other clients continued the therapy until the end. They also collaborated during the follow-up stage. As stated previously, the early drop-out of the last client can be explained in terms of several factors such as high ambivalence, high distress, borderline personality traits, reliance on immature defenses (e.g., denial, repression, splitting), insecure attachment and her partner's ambivalent behaviors. The client's borderline personality traits, as well as her partner's ambivalent behaviors seem to be determinant factors for her early drop-out. The latter factor seems to be more true, because when she was completely rejected by her partner, she came back to begin her therapy again and made a significant progress. Although the majority of the clients accepted this therapy, in order to be able to generalize the findings, further studies with larger sample sizes are needed. Therefore, the results of this study must be interpreted with caution. 

*5) Safety (does the therapy have any adverse effect?):*

The findings of LTI, BDI-II and GAF indicated that the therapy improved the clients' mental health significantly. None of the clients complained about the therapeutic processes. Since no specific instrument was used in the study to assess the clients' satisfaction, the extent of clients' compliance cannot be determined aside from the fact that all of the clients attended the required therapy session. 

*6)* *Stability(how long therapeutic changes last?):*

The results of the study indicated that the changes were held at a two–month follow-up. Although a two-month follow-up is too short to draw definite conclusions regarding the efficacy of the intervention, it can be argued that the intervention has had significant effects, because the improvements had continued even two months after the termination of therapy. Besides, considering the processes of grief, long-lasting changes can be predicted. As has been stated before, grief analysis is considered a guided and tolerable re-enacting of separation-individuation stage of development. Considering the fact that McCullough's model emphasizes extensively on the defenses, affects and self/other restructuring (10,11), the therapy can be a corrective emotional experience for the client by re-enacting his/her past conflictual experiences with different consequences than the past ones (22). 

## Authors’ Contributions

MD conceived and designed the evaluation, collected the clinical data, interpreted them and helped to draft the manuscript. M-K-V participated in designing the evaluation and performed the statistical analysis, and revised the manuscript. BG participated in designing the evaluation, re-analyzed the clinical data and revised the manuscript. All authors read and approved the final manuscript.

## Notes

**Declaration of Interest:** None.

**Clinical trial registration:** URL: http://www.irct.ir/ Unique identifier: IRCT 138903063996N2
